# Compositional Analysis of Polymeric Proanthocyanidins from *Vitis amurensis* Rupr. (Vitaceae) Seeds After Catechin-Assisted Sulfitolytic Cleavage

**DOI:** 10.3390/foods15122045

**Published:** 2026-06-06

**Authors:** Xiangyun Ren, Peixin Wang, Jing Lan, Zhangcheng Liang, Zhigang He, Hao Su, Weixin Li

**Affiliations:** 1 Institute of Food Science and Technology, Fujian Academy of Agricultural Sciences, Fuzhou 350003, China; yuin09@163.com (X.R.); peixin_wang@yeah.net (P.W.); 15980521089@163.com (J.L.); supper3231@163.com (Z.L.); njgzx@163.com (Z.H.); suhaoxp@163.com (H.S.); 2 Fujian Key Laboratory of Agricultural Products (Food) Processing, Fuzhou 350013, China; 3 Key Laboratory of Subtropical Characteristic Fruits, Vegetables and Edible Fungi Processing, Ministry of Agriculture and Rural Affairs, Fuzhou 350013, China

**Keywords:** *Vitis amurensis* seeds, polymeric proanthocyanidins, depolymerization, catechin, sulfitation

## Abstract

Polymeric procyanidins (PPCs) constitute the major fraction of procyanidins, but they have poor bioactivity. The purpose of this study is to clarify the composition and content of PPCs from *Vitis amurensis* Rupr. (Vitaceae) seeds before and after depolymerization, thereby providing a theoretical basis for activity evaluation and application of proanthocyanidins (PCs). PPCs extracted from *V. amurensis* seeds were depolymerized by catechin-assisted sulfitation. The compositions and contents of PCs before and after depolymerization were qualitatively and quantitatively analyzed by ultra-performance liquid chromatography–tandem mass spectrometry (UPLC-MS/MS), high-performance liquid chromatography (HPLC), and liquid chromatography–tandem mass spectrometry (LC-MS/MS). Results showed that twenty-eight components were identified (7 monomers, 13 dimers, 5 trimers, 1 tetramer and 2 unknowns). Before depolymerization, tetrameric and higher polymers dominated, accounting for 58.81% of the relative content. After depolymerization, these high-molecular-weight compounds declined to <1% or became undetectable, while monomers and dimers (with minor trimers) surged to 42.89%. Among them, the relative content of two monomers and three dimers, catechin, epicatechin gallate and procyanidin B1–B3, increased by 37.00, 3.75, 10.98, 3.72 and 9.74 times, respectively. In conclusion, the method utilizing catechin-assisted sulfitation effectively depolymerizes PPCs from *V. amurensis* seeds into oligomeric components such as monomers and dimers.

## 1. Introduction

Proanthocyanidins (PCs), as an important class of flavonoid compounds, have molecular structures formed by the polymerization of monomers such as catechin and epicatechin through carbon–carbon bonds [1]. They possess various biological activities, including antioxidant effects, prevention of cardiovascular diseases, and hypoglycemic properties [2], thus holding significant application value in fields such as pharmaceutical development, food processing, and biochemical engineering. PCs can be categorized into three groups according to their degree of polymerization: monomers, oligomers, and polymers. Monomeric proanthocyanidins (MPCs), with a degree of polymerization (DP) of 1, primarily include four compounds: (+)-catechin (C), (−)-epicatechin (EC), (+)-catechin-3-O-gallate (CG), and (−)-epicatechin-3-O-gallate (ECG) [3]. Oligomeric proanthocyanidins (OPCs), with a DP ranging from 2 to 5, and polymeric proanthocyanidins (PPCs), with a DP greater than 5 and potentially reaching several dozen units, represent the higher polymerized forms [4,5]. Based on the types of intermonomeric linkages and degree of polymerization, proanthocyanidins are systematically categorized. Type B PCs are defined exclusively by C4–C8 or C4–C6 carbon–carbon bonds; among these, dimers B1–B4 (C4–C8 linkage) are the most abundant in fruit-bearing plants, whereas dimers B5–B8 (C4–C6 linkage) occur in lower amounts [6,7]. Type A PCs are characterized by an additional C2–O7 ether linkage beyond the carbon–carbon bond; the dimers A1 and A2 are representative forms found in peanuts, lychees, and cranberries [8]. C-type PCs refer to trimers formed via C4β-C8 bonds between catechin blocks (C1) or via C4α-C8 bonds between epicatechin blocks (C2) [9]. Furthermore, higher oligomers such as tetramers and pentamers may consist exclusively of A-type or B-type linkages, or contain a mixture of both. Oligomeric proanthocyanidins can be rapidly absorbed in the human small intestine, exhibiting a bioavailability exceeding 90%. However, as the degree of polymerization increases, the molecular structure becomes more complex, leading to significantly reduced biological activity and bioavailability [10,11].

Grape seeds, a major byproduct of the winemaking process, contain polyphenolic compounds that constitute approximately 50–70% of the total phenolics in grapes. Among these phenolic extracts, proanthocyanidins constitute as much as 80–85% [12], making grape seeds a significant industrial source for proanthocyanidin extraction. However, in grape seed proanthocyanidins, PPCs account for over 50% of the total. Therefore, depolymerization is required to break down these high-molecular-weight polymers into lower-degree proanthocyanidins, thereby enhancing their bioactivity and bioavailability. Current methods for degrading polymeric proanthocyanidins include acid or base catalysis, flavanol monomer-mediated degradation, enzymatic hydrolysis, and microbial degradation [10,13]. However, these methods often suffer from drawbacks such as poor degradation efficiency, high cost, complex reaction conditions, or the introduction of impurities [14,15]. Among these approaches, catechin-assisted sulfitolytic cleavage has emerged as a promising strategy. It operates under mild conditions with high efficiency and controllability, resulting in fewer impurities compared to traditional techniques [16]. Notably, sulfite is a common additive in wine processing [17]. Utilizing it for depolymerization is not only cost-effective but also significantly safer than using strong acids (such as hydrochloric acid), making it highly suitable for the modification of grape-derived products intended for food applications. For example, Luo et al. [18] previously utilized sulfurous acid to depolymerize PPCs from grape seeds and skins, achieving partial conversion into oligomers. Building upon previous research, the authors investigated and optimized a catechin-assisted sulfite depolymerization process using highly purified polymeric proanthocyanidins extracted from seeds of the *V*. *amurensis* Rupr. (Vitaceae) variety “Ziqiu.” This process reduced the mean degree of polymerization (mDP) by 4.09 units. Moreover, a significant enhancement in antioxidant capacity was observed in the depolymerized products (i.e., oligomeric proanthocyanidins), with DPPH· and ABTS+ scavenging activities increasing by 3.31- and 1.28-fold, respectively, compared to the original polymers [19]. However, due to the unclear composition of the depolymerized products, the specific compounds primarily responsible for the enhanced antioxidant activity remain unidentified. Currently, research on grape seed proanthocyanidins both domestically and internationally mainly focuses on extraction and purification techniques, evaluation of biological activities, and product development [20]. In contrast, studies on the chemical composition and structural characteristics of oligomers generated after depolymerization are relatively scarce, and research using *V. amurensis* seeds as the source material is even more limited.

The aim of this study was to analyze the composition and content of PCs from *V. amurensis* seeds before and after depolymerization via catechin-assisted sulfitolytic cleavage, thereby providing a foundation for understanding the depolymerization process and for further evaluation of the application potential of the resulting OPCs.

## 2. Materials and Methods

### 2.1. Materials

Highly polymeric proanthocyanidins from grape seeds were prepared using seeds of *V*. *amurensis* variety “Ziqiu” provided by Fujian Chunqiu Agriculture and Forestry Technology Co., Ltd. (Quanzhou, China). The preparation process included defatting and ultrasonic-assisted extraction with ethanol solution (60% (*v*/*v*) ethanol as the solvent, with an ultrasonic time of 20 min, ultrasonic power of 80 W, extraction temperature of 45 °C, and a solid-to-liquid ratio of 1:30), followed by purification and isolation, yielding proanthocyanidins with an average degree of polymerization of 5.92. The detailed preparation procedures and characterization data have been described in our previous study [19]. Catechin and vanillin (analytical grade) were supplied by Shanghai Yien Chemical Technology Co., Ltd. (Shanghai, China); methanol, acetonitrile, formic acid, and EDTA-2Na (HPLC grade) were purchased from Thermo Fisher Scientific Inc. (Waltham, MA, USA); catechin, epicatechin, epigallocatechin, epigallocatechin gallate, and epicatechin gallate (HPLC grade) were obtained from Sigma-Aldrich Corporation (St. Louis, MO, USA); proanthocyanidins A1, A2, B1, B2, B3, B4, and C1 (HPLC grade) were supplied by Extrasynthese S.A. (Genay, France); and sodium sulfite and other common reagents (analytical grade) were provided by Sinopharm Chemical Reagent Co., Ltd. (Shanghai, China).

### 2.2. Depolymerization Process of Polymeric Proanthocyanidins

The method described by Luo et al. [18] was referenced and slightly modified as follows: Polymeric proanthocyanidins were dissolved in methanol to prepare a stock solution at a concentration of 10 mg/mL (*w*/*v*). Then, catechin was weighed at a ratio of 0.45:1.0 (catechin: polymeric proanthocyanidins) and added to the stock solution. This optimal ratio was determined by single-factor experiments and response surface methodology in our previous study [19]. The mixture was thoroughly homogenized and heated in a water bath to 40 °C. Then, a sulfite reagent (SO_2_ ≥ 6.0%) was added to the mixture at a concentration of 6% (*v*/*v*). After immediate shaking to ensure uniform mixing, the reaction mixture was transferred to a thermostatic shaking water bath set at 40 °C and 200 rpm for 31 min. Upon completion, the mixture was removed and cooled. The reaction was terminated by adding 0.1 mol/L NaOH–methanol solution dropwise under gentle stirring until the pH reached 7.0 (monitored with a pH meter). After dilution, the average degree of polymerization was determined.

### 2.3. Qualitative Analysis of Proanthocyanidin Components in Samples Before and After Depolymerization by UPLC-MS/MS

The method of sample extraction described by He et al. [21] was used. The UPLC system was a LC-30 (Shimadzu Corporation, Kyoto, Japan), and the separation was achieved on an ACQUITY UPLC HSS T3 column (1.8 µm, 2.1 mm × 100 mm; Waters Corporation, Milford, MA, USA). The UPLC parameters were as follows: 2 μL injection volume, flow rate of 0.3 mL/min, column temperature of 50 °C. The UPLC gradient elution program was as follows: 0–0.5 min, 5% A (0.1% formic acid in water) and 95% B (0.1% formic acid in acetonitrile); 0.5–2.5 min, B decreased linearly from 95% to 70%; 2.5–7.5 min, B increased linearly from 70% to 100%; 7.5–9.0 min, maintained at 100% B; 9.0–9.5 min, B decreased from 100% to 5% for column re-equilibration; 9.5–12.0 min, maintained at 5% B. The eluted analytes were detected using a TripleTOF 5600+ high-resolution mass spectrometer (SCIEX, Maastricht, The Netherlands) equipped with an electrospray ionization (ESI) source [22]. The mass spectrometer was operated in both positive and negative ion modes. The ESI source conditions were set as follows: Ion Source Gas 1 (Gas 1): 60, Ion Source Gas 2 (Gas 2): 60, Curtain Gas (CUR): 30, source temperature: 600 °C, IonSapary Voltage Floating (ISVF): ±5500 V. The mass spectrometry parameters included: TOF MS scan *m*/*z* range: 60–1200 Da, product ion scan *m*/*z* range: 25–1200 Da, TOF MS scan accumulation time: 0.15 s/spectrum, and product ion scan accumulation time: 0.03 s/spectrum. Information Dependent Acquisition (IDA) was employed to obtain secondary mass spectra in high-sensitivity mode. The specific IDA parameters were: Declustering Potential (DP): ±60 V, collision energy: 30 eV, exclude isotopes within 4 Da, and candidate ions to monitor per cycle: 6.

### 2.4. Quantification of Monomeric Proanthocyanidin Components Before and After Depolymerization

HPLC was used for quantitative analysis of monomeric proanthocyanidins, following the method for catechins specified in the Chinese National Standard GB/T 8313 [23], “Determination of tea polyphenols and catechins in tea.” The target analytes included catechin, epicatechin, epigallocatechin, epigallocatechin gallate, and epicatechin gallate.

#### 2.4.1. Chromatographic Conditions

The HPLC system was a Waters 2695 (Waters Corporation, Milford, MA, USA). The separation was performed on an Agilent ZORBAX SB-C18 column (5 μm, 250 mm × 4.6 mm; Agilent Technologies Inc., Santa Clara, CA, USA). Mobile phase A was prepared by mixing 90 mL acetonitrile, 20 mL acetic acid, and 2 mL of 10 mg/mL EDTA-2Na solution, then diluted to 1000 mL with water. Mobile phase B was prepared by mixing 800 mL acetonitrile, 20 mL acetic acid, and 2 mL of 10 mg/mL EDTA-2Na solution, then diluted to 1000 mL with water. The HPLC conditions were as follows: flow rate of 1 mL/min, column temperature of 35 °C, detection wavelength at 278 nm, and injection volume of 10 μL. The gradient elution program was as follows: 0–10 min, 100% A (aqueous phase); 15–25 min, linearly changed to 68% A and 32% B (organic phase); 25–30 min, returned to 100% A for column re-equilibration.

#### 2.4.2. Quantification of MPCs

Quantification was performed using peak area. The linear equations and correlation coefficients of the standard curves for the monomeric components are shown in Table 1. The content of the proanthocyanidin monomer was calculated using the following equation:

(1) $$c\;=\;\frac{(A-A_{0})\times f_{\mathrm{std}}\times V\times d\times 100}{m\times 10^{6}\times \omega }$$

where c—content of the proanthocyanidin monomer, %; A—peak area of the target compound in the sample; A_0_—peak area of the corresponding compound in the reagent blank; F_Std_—correction factor (concentration/peak area) of the measured compound, g/mL; V—total volume of the sample extract, mL; m—mass of the sample taken, g; *ω*—dry basis content (mass fraction) of the sample, %; d—dilution factor.

### 2.5. Determination of Oligomeric Proanthocyanidin Components Before and After Depolymerization

For quantitative analysis of oligomeric proanthocyanidins, the following compounds were selected: proanthocyanidin A1, A2, B1, B2, B3, B4, and C1. The analysis was performed using LC-MS/MS according to the method described by Hu et al. [24,25]. The LC-MS/MS system consisted of an ExionLC liquid chromatograph (SCIEX, Framingham, MA, USA) and a QTRAP 6500+ mass spectrometer (SCIEX, Framingham, MA, USA). Chromatographic separation was achieved on an ACQUITY BEH C18 column (1.7 µm, 2.1 mm × 100 mm; Waters Corporation, Milford, MA, USA).

#### 2.5.1. Chromatographic and Mass Spectrometric Conditions

Liquid chromatography conditions: The mobile phase was composed of solvent A (0.5% formic acid in water prepared with ultrapure water) and solvent B (methanol containing 0.5% formic acid). The gradient elution program was as follows: 0 min, 5% B; 6 min, increased to 50% B; 12 min, increased to 95% B; held for 2 min; 14 min, decreased to 5% B, followed by equilibration for 2 min. The flow rate was 0.35 mL/min, the column temperature was maintained at 40 °C, and the injection volume was 2 μL.

Mass spectrometry parameters: Electrospray ionization (ESI) source temperature: 550 °C; ion spray voltage: 5500 V in negative ion mode; curtain gas pressure: 35 psi. Analyte identification was not based solely on nominal mass. An in-house library was constructed using standards for qualitative analysis of mass spectrometry data. Quantification was performed using multiple reaction monitoring (MRM) mode: the first quadrupole selects precursor ions to remove interference; precursor ions are fragmented in the collision cell; and the third quadrupole filters characteristic fragment ions, eliminating non-target ions for more accurate and reproducible quantification. After data acquisition, chromatographic peaks of all targets were integrated and quantified via a standard curve.

#### 2.5.2. Quantitative Analysis of Oligomeric Proanthocyanidins

Standard working curves for each target compound were constructed by plotting the series concentrations of the standards as the *x*-axis against the corresponding chromatographic peak areas as the *y*-axis. The linear equations and correlation coefficients for the oligomeric components are presented in Table 2.

The content of the target substances in the actual samples is determined by substituting the integrated chromatographic peak area values of the detected samples into the linear equation of the standard curve. After calculation using the quantitative formula, the accurate content of the target substances in the samples is obtained.

(2) $$M=\frac{c\times V}{1,000,000\times m}$$

In the formula, M—the content of the metabolite in the sample, μg/g; c—the concentration value obtained by substituting the peak area into the standard curve, ng/mL; V—the volume of extraction solution used, μL; m—the mass of the sample, g.

### 2.6. Statistical Analysis

Experimental data were processed and statistically analyzed using DPS 6.01.

## 3. Results

### 3.1. Qualitative Analysis of Proanthocyanidin Components Before and After Depolymerization

The total ion chromatograms (TIC) of the samples before and after depolymerization in positive and negative ion modes are shown in Supplementary Materials (Figures S1 and S2). A total of 190 flavonoids were detected in the proanthocyanidins from *V. amurensis* seeds, including 28 proanthocyanidins: 7 monomers, 13 dimers, 5 trimers, 1 tetramer, and 2 unidentified proanthocyanidins (Table 3). The mDP of highly polymerized proanthocyanidins from *V. amurensis* seeds, after depolymerization using catechin-assisted sulfite cleavage, was determined to be 1.83 [19]. Compared with the original PPCs, most components in the depolymerized product—OPCs—increased in relative abundance, while a few decreased.

Among the monomeric components, catechin exhibited the greatest increase, followed by epicatechin gallate. Their relative contents in OPCs reached 33.31% and 2.94%, respectively, representing 26.65-fold and 5.34-fold increases compared to those in PPCs. Among the dimers, procyanidin B2, B3, and B-type procyanidin dimers showed significant increases in relative content, followed by procyanidin B5 and B1. Their relative contents in OPCs were 2.63%, 1.54%, 0.11%, 0.77%, and 1.40%, respectively, which were 12.98-, 11.51-, 10.15-, 8.70-, and 5.23-fold higher than in PPCs. Notably, two dimeric proanthocyanidins (No. 12 and No. 19) were only detected in OPCs, suggesting they were newly formed during the depolymerization process. Among the trimers, procyanidin C1 and C2 showed significant increases, with relative contents in OPCs 8.70- and 10.51-fold higher than in PPCs, respectively.

In OPCs, the relative contents of two monomers—gallocatechin gallate and epigallocatechin gallate—and three dimers decreased, possibly due to their conversion into other isomers during the depolymerization process. The relative content of the tetrameric proanthocyanidin significantly decreased in OPCs, likely due to its breakdown into lower-degree oligomers. Although the complex structure of PPCs makes direct separation and identification difficult, two unidentified components showed high relative abundances in PPCs (33.07% and 16.93%) but were nearly undetectable in OPCs. This suggests that these two components were highly polymerized proanthocyanidins that were converted into lower oligomers during depolymerization.

During the conversion of PPCs to OPCs, the relative content of components with a degree of polymerization ≥ 4 decreased from 58.81% to less than 1% or became undetectable, while the combined relative content of monomers, dimers, and trimers increased from 2.56% to 42.89%. These results indicate that the catechin-assisted sulfite degradation method used in this study effectively converts highly polymerized proanthocyanidins into low-polymerization oligomers with higher biological activity.

### 3.2. Quantitative Analysis of Monomeric Components in Proanthocyanidins Before and After Depolymerization

**Figure 1:**
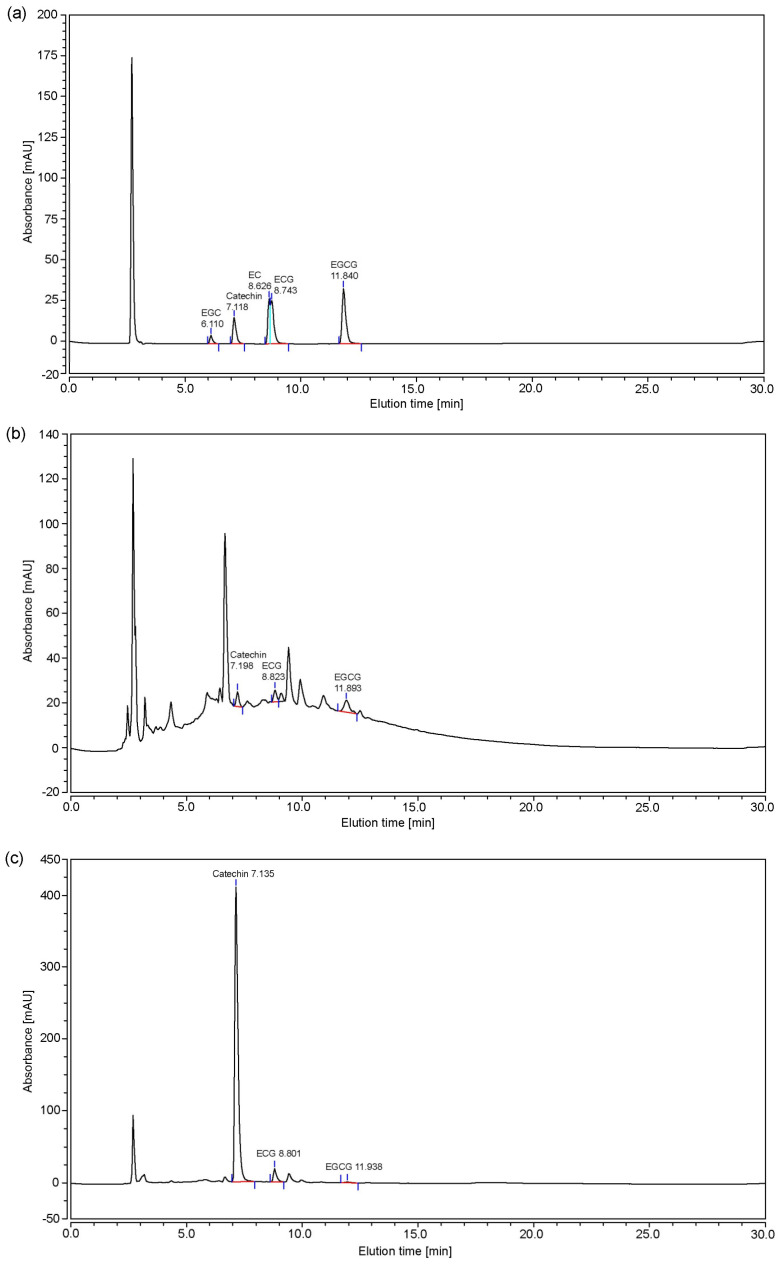
HPLC chromatograms of MPCs in PCs before and after depolymerization. (**a**) Mixed standard; (**b**) Before depolymerization; (**c**) After depolymerization.

The HPLC chromatograms and contents of monomeric components in PPCs before and after depolymerization are shown in Figure 1 and Table 4. Compared with the standard compounds (Figure 1a), the chromatogram of PPCs before depolymerization (Figure 1b) exhibited multiple broad peaks between 2–8 min, suggesting the presence of other polyphenolic substances in the PPC material that are difficult to separate. In addition, the PPC raw material contained small amounts of monomers, with retention times at t = 7.198, 8.823, and 11.893 min corresponding to catechin, epicatechin gallate, and epigallocatechin gallate, respectively, with concentrations of 1.80 × 10^3^, 8.00 × 10^2^, and 1.30 × 10^3^ μg/g.

In the chromatogram of the depolymerized product (OPCs) (Figure 1c), no unresolved broad peaks were observed between 2–8 min, and the detected monomer peaks matched those present in PPCs, indicating that no new monomers were generated during depolymerization. However, significant changes in their concentrations were observed (Table 4). The content of catechin reached 1.21 × 10^5^ μg/g; after subtracting the amount added as a nucleophilic reagent, the residual catechin content was still 6.66 × 10^4^ μg/g, representing a 37-fold increase compared to the catechin content in PPCs. This indicates that the depolymerization method used in this study effectively degraded PPCs into catechin monomers. Additionally, the content of epicatechin gallate reached 3.00 × 10^3^ μg/g, which is 3.75 times higher than that in PPCs. While these two components increased, the content of epigallocatechin gallate decreased by one order of magnitude, likely due to its conversion into other isomers (other monomeric forms) during the depolymerization reaction [26].

### 3.3. Quantitative Analysis of Oligomeric Components in Proanthocyanidins Before and After Depolymerization

**Figure 2:**
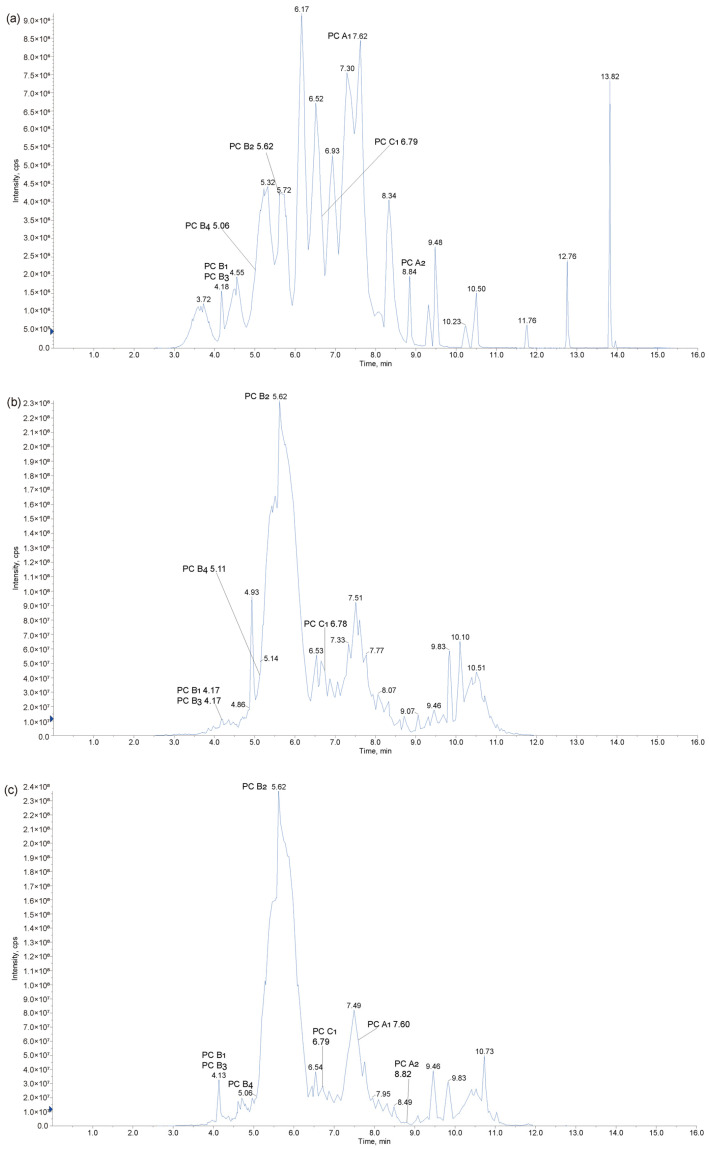
Ion chromatogram of OPCs. (**a**) Mixed standard; (**b**) Before depolymerization; (**c**) After depolymerization.

The MS/MS spectra of oligomeric proanthocyanidin standards obtained from library construction are presented in the Supplementary Materials (Figures S3–S9). The extracted ion chromatograms and contents of oligomeric proanthocyanidins before and after PPC depolymerization are shown in Figure 2 and Table 5. A total of seven oligomeric proanthocyanidin components were detected in OPCs, including six dimers and one trimer.

Compounds numbered 1 and 2 had retention times of 7.61 min and 8.85 min, respectively, with *m*/*z* values of 575.1 and 575.3—each 2 Da lower than typical B-type dimers—indicating the presence of an additional C–O–C bond in their structures, consistent with the fragmentation pattern of A-type dimers. By comparison with reference standards, these were identified as procyanidin A1 and A2, respectively. These two compounds were only detected in OPCs, suggesting they were newly formed during the depolymerization process, although their contents were low at 0.08 μg/g and 0.31 μg/g, respectively.

Compounds numbered 3, 4, 5, and 6 had retention times of 4.18 min, 5.62 min, 4.18 min, and 5.12 min, respectively, with *m*/*z* values all at 577.1. Based on mass spectral analysis, these compounds are likely B-type procyanidin dimers, with the *m*/*z* value corresponding to the characteristic [M–H]^−^ ion formed under ESI^−^ conditions. Comparison with standards confirmed their identities as procyanidin B1, B2, B3, and B4. After depolymerization, the contents of all four dimers increased significantly, ranging from 7.87 to 50.53 μg/g. Specifically, procyanidin B1 increased 10.98-fold, while B2, B3, and B4 increased 3.72-, 9.74-, and 5.63-fold, respectively.

Compound number 7 had a retention time of 6.78 min and an *m*/*z* value of 865.2. Comparison with the standard confirmed it as the trimeric procyanidin C1. Its content in OPCs was 6.48 μg/g, representing a 6.29-fold increase compared to the level before depolymerization.

## 4. Discussion

In the present study, catechin-assisted sulfitolytic cleavage was employed to prepare OPCs from PPCs of *V. amurensis* seeds. The use of sulfite (SO_3_^2−^) in the depolymerization reaction is critical for cleaving the interflavan C–C bonds of polymeric proanthocyanidins. Under acidic conditions, sulfite acts as a nucleophile and attacks the electrophilic C4 position of the extension units, leading to the formation of a flavan-4β-sulfonate intermediate [27]. This intermediate is unstable and rapidly undergoes further transformation in the presence of catechin (as a trapping nucleophile), resulting in the release of lower-molecular-weight oligomers, predominantly monomers and dimers. The sulfitolysis mechanism is well established for proanthocyanidin depolymerization and offers higher selectivity and milder reaction conditions compared to conventional acid-catalyzed degradation [28].

The structure of proanthocyanidins in grape seeds is highly complex, characterized by two main features: first, the number of isomers follows an exponential relationship of 2^n−1^ × 3^n^ (where n = degree of polymerization), increasing dramatically with polymerization degree; second, different oligomers and their homologous isomers have similar polarities, making effective separation by conventional liquid chromatography challenging [29,30]. As a result, only a limited number of isomers can typically be isolated, posing significant difficulties in the analysis and purification of grape seed proanthocyanidins [31,32,33]. In this study, UPLC-MS/MS was employed to identify the compositional profile and relative abundances of PPCs from grape seeds before and after depolymerization. Based on this, selected standard compounds were used to quantify specific monomeric and oligomeric components via HPLC and LC-MS/MS.

Qualitative analysis revealed 28 proanthocyanidin-related components in *V. amurensis* seeds, including 7 monomers, 13 dimers, 5 trimers, 1 tetramer, and 2 unidentified components, which are presumed to be highly polymerized structures. Prior to depolymerization, the proanthocyanidin composition was dominated by tetramers and higher polymers, with a combined relative content of 58.81%, while monomers, dimers, and trimers together accounted for only 2.56%. After depolymerization, the relative content of components with a polymerization degree ≥ 4 dropped to below 1% or became undetectable. This decrease was accompanied by a significant increase in monomers, dimers, and a small amount of trimers, whose total relative content rose to 42.89%. Quantitative analysis showed that two monomers (catechin, epicatechin gallate) and three dimers (procyanidin B1–B3) with relative contents exceeding 1% increased by 37.0-, 3.75-, 10.98-, 3.72-, and 9.74-fold, respectively, in the depolymerized product (OPCs). It should be noted that catechin was externally added as a nucleophile to facilitate the sulfitolysis reaction. Therefore, the total catechin detected after depolymerization includes both the exogenously added catechin and the catechin released from PPCs. By conducting a blank control experiment without PPCs, the exogenous contribution was quantified and subtracted. The net catechin derived from depolymerization (6.66 × 10^4^ μg/g) was still 37-fold higher than the endogenous catechin level in the original PPCs (1.80 × 10^3^ μg/g). This confirms that the observed increase is not an artifact of external addition but rather a true measure of depolymerization efficiency.

Existing studies have demonstrated that catechin, as a typical flavonoid, exhibits significant antibacterial, antioxidant, and anticancer activities [34,35]. Epicatechin, an important dietary flavonoid, has well-documented effects in regulating blood pressure, improving lipid metabolism, and maintaining glucose homeostasis [36]. Procyanidin B2, a dimeric proanthocyanidin, has been shown to modulate gut microbiota and exert antitumor effects [37]. Additionally, proanthocyanidins have been reported to promote osteogenesis, protect tissue cells, and accelerate wound healing [38]. Moreover, the bioactivity of proanthocyanidins is closely related to their degree of polymerization, with dimers and trimers generally exhibiting the strongest biological activities, followed by monomers and higher oligomers [39,40]. In this study, the depolymerization of high-polymer proanthocyanidins from *V. amurensis* seeds generated substantial amounts of monomers and dimers, indicating that the resulting OPCs possess high bioactivity and enhanced bioavailability, thus offering broad application potential. Furthermore, this confirms that the catechin-assisted sulfite depolymerization method is an effective approach for the oligomerization of grape seed proanthocyanidins.

It is important to compare our results with those of Luo et al. [18], given the similarity in our research topics, specifically the use of sulfurous acid to degrade grape seed PPCs into oligomers. In that study, the authors focused on preparative isolation of individual procyanidin dimers and trimers by HSCCC and prep-HPLC, achieving high yields of compounds such as catechin, epicatechin, procyanidin B1–B4 and C1, and galloylated dimers. However, several key aspects were not addressed: (i) the quantitative changes in the entire oligomer distribution (including tetramers and higher polymers) before and after degradation; (ii) the relative abundance of each component; (iii) the formation of A-type dimers or new B-type dimers; and (iv) the direct linkage between compositional shift and bioactivity enhancement. The present study complements and extends the work of Luo et al. by providing a detailed compositional profile of *V. amurensis* seed PPCs and their depolymerized products using catechin-assisted sulfitolysis cleavage. Furthermore, our method incorporates catechin as a nucleophilic trapping agent, which not only improves degradation efficiency but also leads to a marked increase in catechin monomers and several bioactive dimers. Thus, while Luo et al. established the feasibility of sulfite-based degradation, our work provides a quantitative compositional basis for understanding the bioactivity of the resulting OPCs and demonstrates the additional benefits of catechin assistance.

In previous work, the authors have already established the antioxidant activity of oligomeric proanthocyanidins from *V. amurensis* seeds. Future studies should further evaluate their potential efficacy in lowering blood pressure, regulating blood lipids, and inhibiting tumor growth.

## 5. Conclusions

This study successfully characterized the compositional changes in proanthocyanidins from *V. amurensis* seeds before and after depolymerization. The results demonstrate that catechin-assisted sulfitation is an effective method for breaking down high-polymer proanthocyanidins, which are predominantly tetramers and larger polymers (58.81% relative content), into lower-molecular-weight oligomers. Following depolymerization, the content of high-degree polymers (degree of polymerization ≥ 4) decreased to negligible or undetectable levels, while the relative abundance of bioactive monomers and dimers (particularly catechin, epicatechin gallate, and procyanidins B1–B3) increased significantly. This shift resulted in a substantial rise in the total proportion of monomers, dimers, and trimers to 42.89%. Given that lower-degree oligomers such as dimers and trimers generally exhibit superior bioavailability and biological activity, including antioxidant, antihypertensive, lipid-regulating, and antitumor effects, the obtained OPCs show great promise for functional food and nutraceutical applications. This work provides both a practical depolymerization strategy and a solid theoretical foundation for the enhanced utilization of grape seed proanthocyanidins. Future studies should focus on evaluation of their bioactivities, elucidation of the underlying molecular mechanisms, and development of functional products incorporating the depolymerized oligomeric proanthocyanidins.

## Figures and Tables

**Table 1:** Linear equations and correlation coefficients of the standard curves for MPCs.

Compound	Retention Time (min)	Linear Equation	R^2^
Epigallocatechin (EGC)	6.11	y = 0.0370x − 0.0042	1.0000
Catechin (C)	7.12	y = 0.1213x − 0.0112	1.0000
Epicatechin (EC)	8.62	y = 0.1622x − 0.0664	0.9998
Epicatechin gallate (ECG)	8.74	y = 0.2108x + 0.1317	0.9998
Epigallocatechin gallate (EGCG)	11.84	y = 0.2944x + 0.0170	1.0000

**Table 2:** Linear equations and correlation coefficients of the standard curves for OPCs.

Compound	Retention Time (min)	Linear Equation	R^2^
Proanthocyanidin A1	7.61	y = 91,545.60000x − 23,677.33477	0.99886
Proanthocyanidin A2	8.85	y = 64,385.70000x − 67,156.30000	0.99954
Proanthocyanidin B1	4.18	y = 14,719.49361x + 3337.64044	0.99662
Proanthocyanidin B2	5.62	y = 14,374.42205x + 29,016.51447	0.99140
Proanthocyanidin B3	4.18	y = 14,074.66887x − 3534.86246	0.99767
Proanthocyanidin B4	5.12	y = 27,116.86200x + 22,984.25876	0.99640
Proanthocyanidin C1	6.78	y = 11,600.43543x − 31,075.54000	0.99512

**Table 3:** Components and relative content of PCs before and after depolymerization.

No.	Components		*m*/*z*	*m*/*z* (Calc.)	Δ *m*/*z* (ppm)	Molecular Formula	Relative Content (%)	
							Before Depolymerization	After Depolymerization
1	Monomer	Catechin	291.0862	291.0863	−0.3	C_15_H_14_O_6_	1.24995	33.30737
2		Epigallocatechin	341.0441	341.044	0.3	C_15_H_14_O_7_	0.00018	0.00083
3		Catechin gallate	443.0977	443.0973	0.9	C_22_H_18_O_10_	0.00368	0.00371
4		Epicatechin	289.0716	289.0712	1.4	C_15_H_14_O_6_	0.00093	0.00121
5		Gallocatechin gallate	441.082	441.0816	0.9	C_22_H_18_O_11_	0.01193	0.00055
6		Epigallocatechin gallate	457.0776	457.077	1.3	C_22_H_18_O_11_	0.00211	0.00209
7		Epicatechin gallate	443.0965	443.0973	−1.8	C_22_H_18_O_10_	0.55015	2.93887
8	Dimer	B-type proanthocyanidin dimer	579.1503	579.1497	1	C_30_H_26_O_12_	0.01077	0.10935
9		Proanthocyanidin A1	575.1207	575.119	3	C_30_H_24_O_12_	0.00091	0.00181
10		Proanthocyanidin B2	559.1263	559.124	4.1	C_30_H_26_O_12_	0.20270	2.63119
11		Proanthocyanidin B5	729.1492	729.1454	5.2	C_30_H_26_O_13_	0.08856	0.77006
12		Proanthocyanidin dimer	731.1616	731.1607	1.2	C_37_H_30_O_16_	0.00000	0.00050
13		Proanthocyanidin B3	579.1501	579.1497	0.7	C_30_H_26_O_12_	0.13354	1.53644
14		Proanthocyanidin B1	593.1297	593.129	1.2	C_30_H_26_O_12_	0.26848	1.40307
15		Proanthocyanidin dimer	593.1315	593.1294	3.5	C_30_H_26_O_13_	0.01169	0.00205
16		Proanthocyanidin dimer	561.1396	561.1391	0.9	C_30_H_26_O_13_	0.00222	0.00000
17		Proanthocyanidin dimer	727.1333	727.1299	4.7	C_37_H_30_O_17_	0.00070	0.00085
18		Proanthocyanidin dimer	593.1317	593.1294	3.9	C_30_H_26_O_13_	0.00281	0.00482
19		Proanthocyanidin dimer	563.1555	563.1548	1.2	C_30_H_26_O_13_	0.00000	0.07675
20		Proanthocyanidin dimer	577.1349	577.1341	1.4	C_30_H_26_O_13_	0.01205	0.00569
21	Trimer	Proanthocyanidin C1	865.2018	865.198	4.4	C_45_H_38_O_18_	0.00804	0.06998
22		Proanthocyanidin C2	867.2139	867.213	1	C_45_H_38_O_18_	0.00140	0.01472
23		Proanthocyanidin trimer	860.4647	860.4658	−1.3	C_45_H_36_O_18_	0.00011	0.00053
24		Proanthocyanidin trimer	867.2125	867.213	−0.6	C_45_H_38_O_18_	0.00010	0.00064
25		Proanthocyanidin trimer	861.4511	861.4501	1.2	C_45_H_36_O_18_	0.00123	0.00373
26	Tetramer	Proanthocyanidin tetramer	1155.276	1155.277	−0.9	C_60_H_50_O_24_	8.81148	0.61392
27	Unknown	Unknown	1443.3418	1443.341	0.6	C_75_H_62_O_30_	33.06544	0.00000
28	Unknown	Unknown	1731.4041	1731.405	−0.5	C_90_H_74_O_36_	16.93257	0.00000

**Table 4:** Contents of MPCs in PCs before and after depolymerization.

No.	Components	Before Depolymerization (μg/g)	After Depolymerization (μg/g)
1	Catechin	1.80 × 10^3^	1.21 × 10^5^
2	Epicatechin	—	—
3	Epigallocatechin	—	—
4	Epigallocatechin gallate	1.30 × 10^3^	2.00 × 10^2^
5	Epicatechin gallate	8.00 × 10^2^	3.00 × 10^3^

Note: “—” indicates values not available.

**Table 5:** Contents of OPCs in PCs before and after depolymerization.

No.	Components	Retention Time (min)	Molecular Mass	Molecular Formula	*m*/*z*	Before Depolymerization (μg/g)	After Depolymerization (μg/g)
1	Proanthocyanidin A1	7.61	576.13	C_30_H_24_O_12_	575.1	Not detected	0.08
2	Proanthocyanidin A2	8.85	576.13	C_30_H_24_O_12_	575.3	Not detected	0.31
3	Proanthocyanidin B1	4.18	578.14	C_30_H_26_O_12_	577.1	4.36	47.86
4	Proanthocyanidin B2	5.62	578.14	C_30_H_26_O_12_	577.1	13.59	50.53
5	Proanthocyanidin B3	4.18	578.14	C_30_H_26_O_12_	577.1	4.66	45.38
6	Proanthocyanidin B4	5.12	578.14	C_30_H_26_O_12_	577.1	0.95	7.87
7	Proanthocyanidin C1	6.78	866.21	C_45_H_38_O_18_	865.2	1.40	6.48

## Data Availability

The original contributions presented in this study are included in the article/Supplementary Materials. Further inquiries can be directed to the corresponding author.

## Supplementary Materials

The following supporting information can be downloaded at: https://www.mdpi.com/article/10.3390/foods15122045/s1, Figure S1: Total ion chromatogram of the samples in positive ion mode. (a) Before depolymerization; (b) After depolymerization. Figure S2: Total ion chromatogram of the samples in negative ion mode. (a) Before depolymerization; (b) After depolymerization. Figure S3: The MS/MS spectra of proanthocyanidin A1 obtained from the library construction. Figure S4: The MS/MS spectra of proanthocyanidin A2 obtained from the library construction. Figure S5: The MS/MS spectra of proanthocyanidin B1 obtained from the library construction. Figure S6: The MS/MS spectra of proanthocyanidin B2 obtained from the library construction. Figure S7: The MS/MS spectra of proanthocyanidin B3 obtained from the library construction. Figure S8: The MS/MS spectra of proanthocyanidin B4 obtained from the library construction. Figure S9: The MS/MS spectra of proanthocyanidin C1 obtained from the library construction.

## Abbreviations

The following abbreviations are used in this manuscript:

PCs	Proanthocyanidins
MPCs	Monomeric proanthocyanidins
DP	Degree of polymerization
C	(+)-catechin
EC	(−)-epicatechin
CG	(+)-catechin-3-O-gallate
ECG	(−)-epicatechin-3-O-gallate
OPCs	Oligomeric proanthocyanidins
PPCs	Polymeric proanthocyanidins
UPLC-MS/MS	Ultra-performance liquid chromatography–tandem mass spectrometry
HPLC	High-performance liquid chromatography
LC-MS/MS	Liquid chromatography–tandem mass spectrometry
ESI	Electrospray ionization
MRM	Multiple reaction monitoring
mDP	Mean degree of polymerization
TIC	Total ion chromatograms
