# Supplementary material for: Compositional Analysis of Polymeric Proanthocyanidins from Vitis amurensis Rupr. (Vitaceae) Seeds After Catechin-Assisted Sulfitolytic Cleavage
Source: Foods. 2026 Jun 6;15(12):2045. doi: 10.3390/foods15122045 (PMC13298973; doi:10.3390/foods15122045)
Supplement: Supplementary file 1 [file foods-15-02045-s001.zip › foods-4259318-supplementary.pdf]

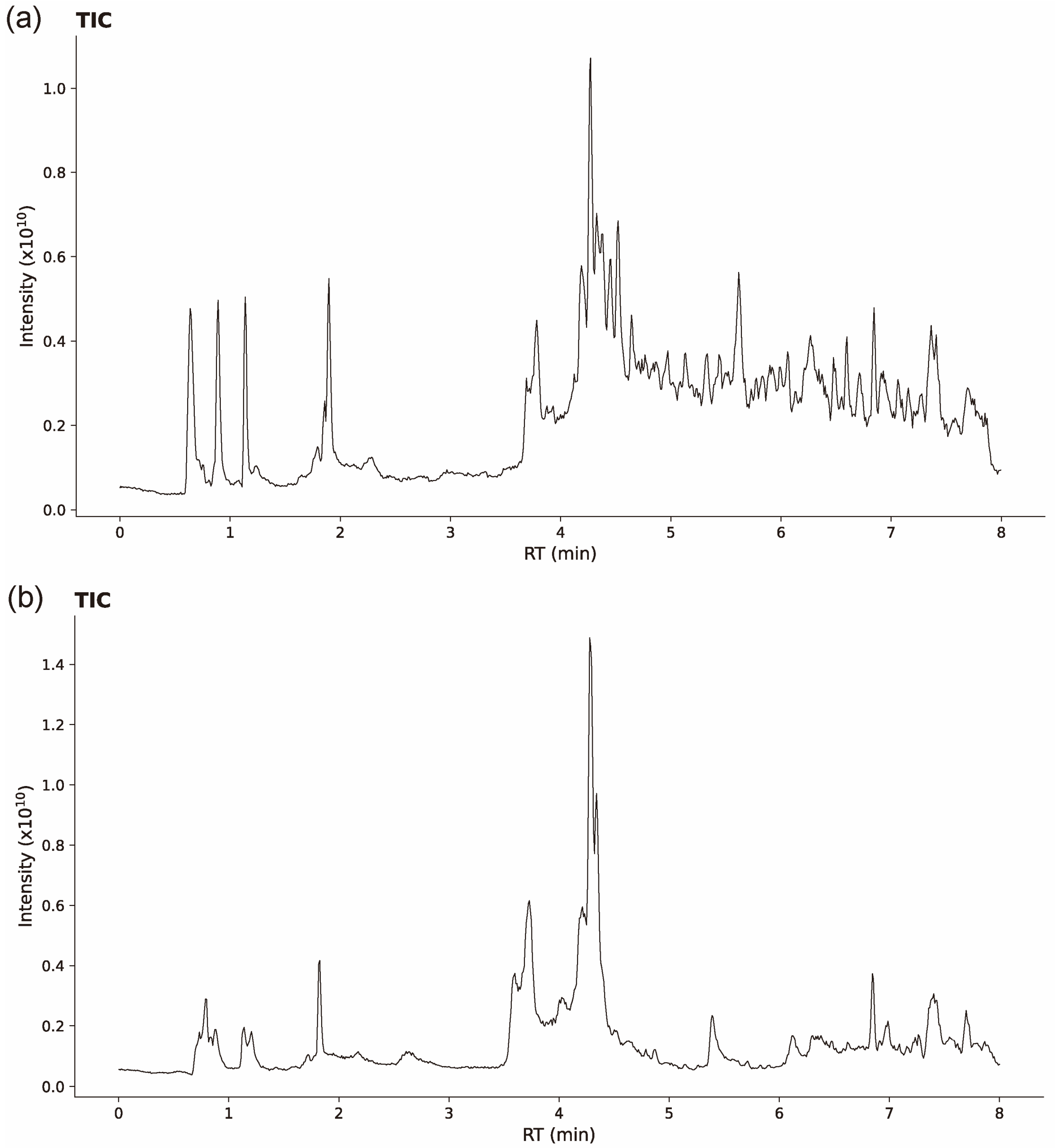

Figure S1 Total ion chromatogram of the samples in positive ion mode. (a) Before depolymerization; (b) After depolymerization

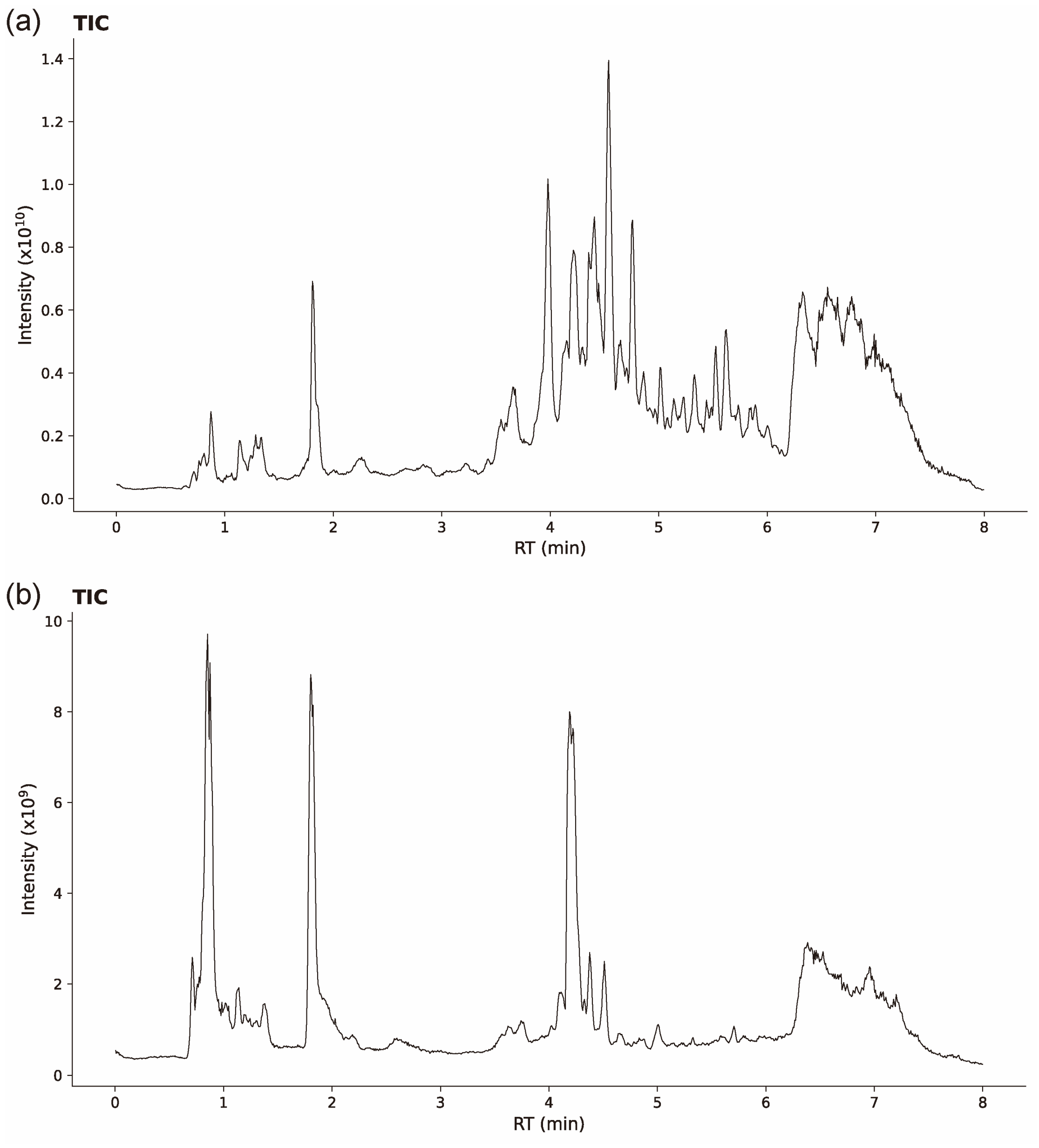

Figure S2 Total ion chromatogram of the samples in negative ion mode. (a) Before depolymerization; (b) After depolymerization

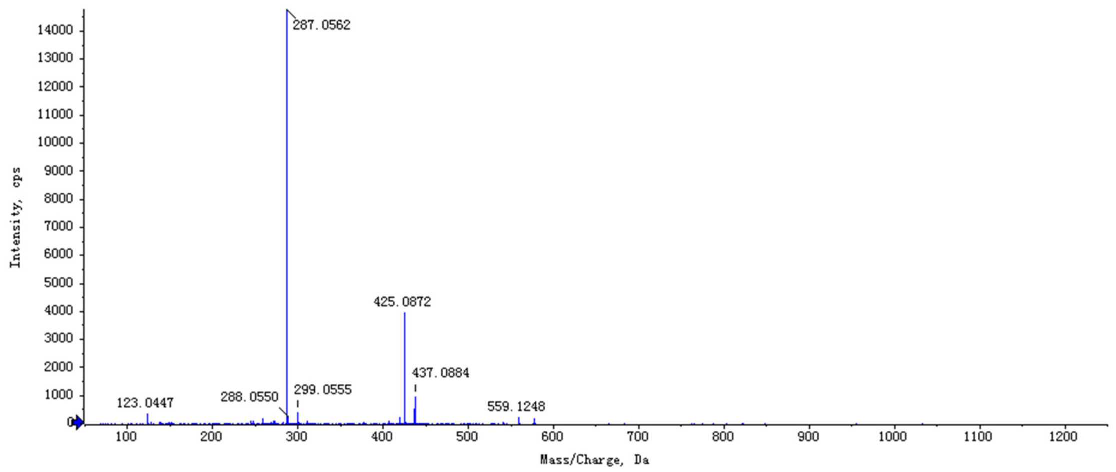

Figure S3 The MS/MS spectra of proanthocyanidin A1 obtained from the library construction

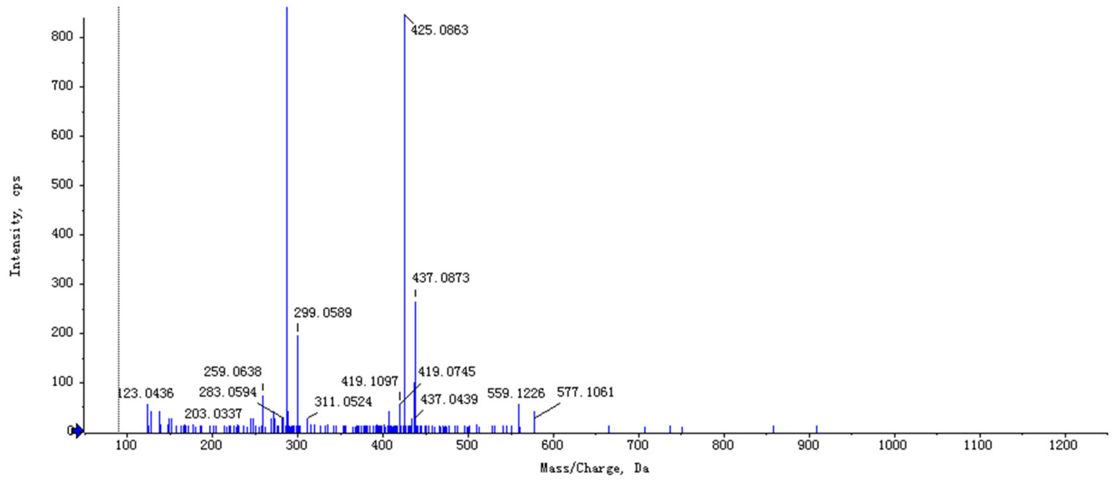

Figure S4 The MS/MS spectra of proanthocyanidin A2 obtained from the library construction

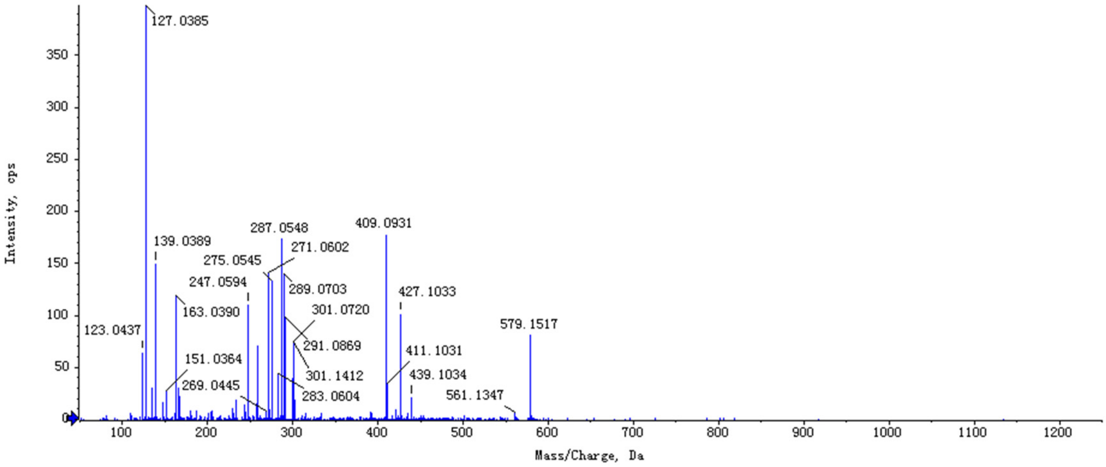

Figure S5 The MS/MS spectra of proanthocyanidin B1 obtained from the library construction

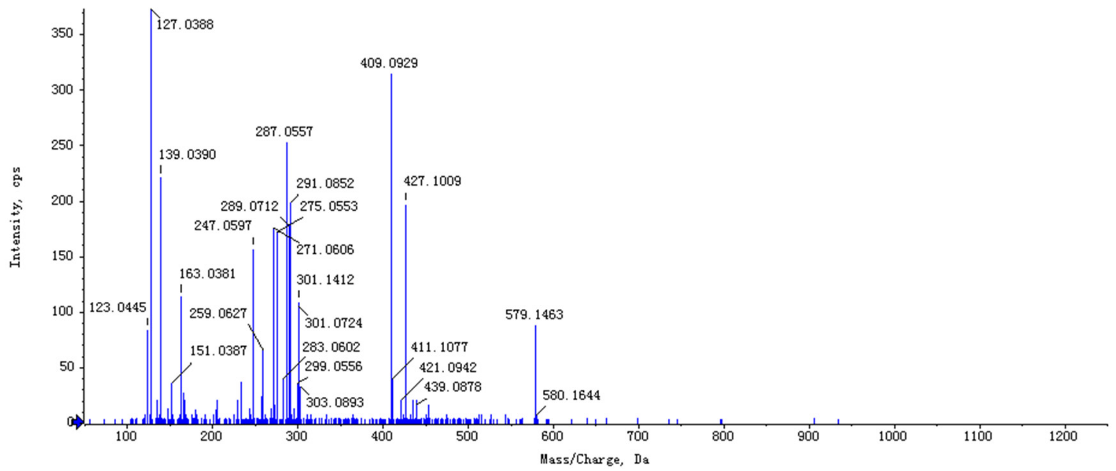

Figure S6 The MS/MS spectra of proanthocyanidin B2 obtained from the library construction

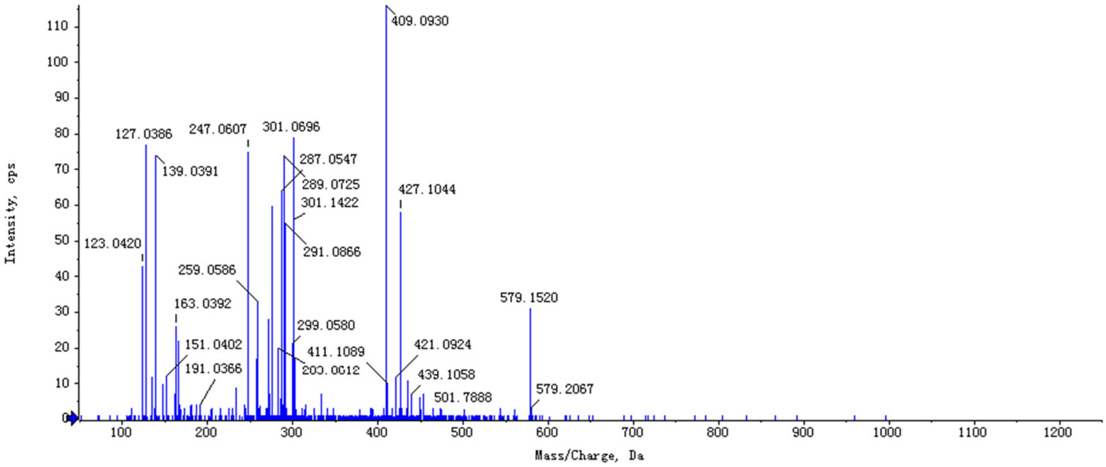

Figure S7 The MS/MS spectra of proanthocyanidin B3 obtained from the library construction

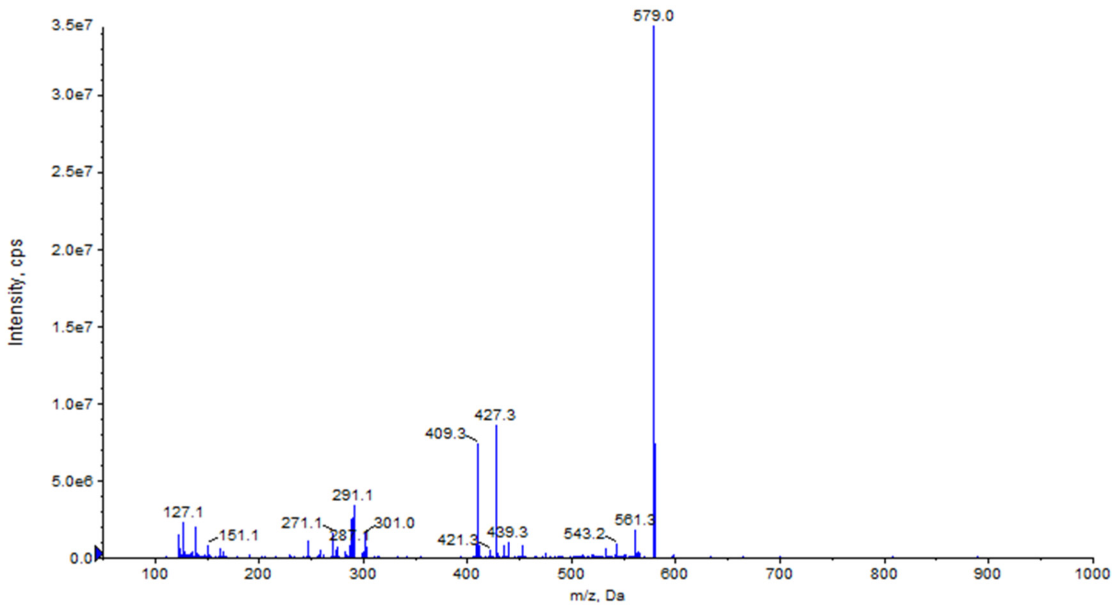

Figure S8 The MS/MS spectra of proanthocyanidin B4 obtained from the library construction

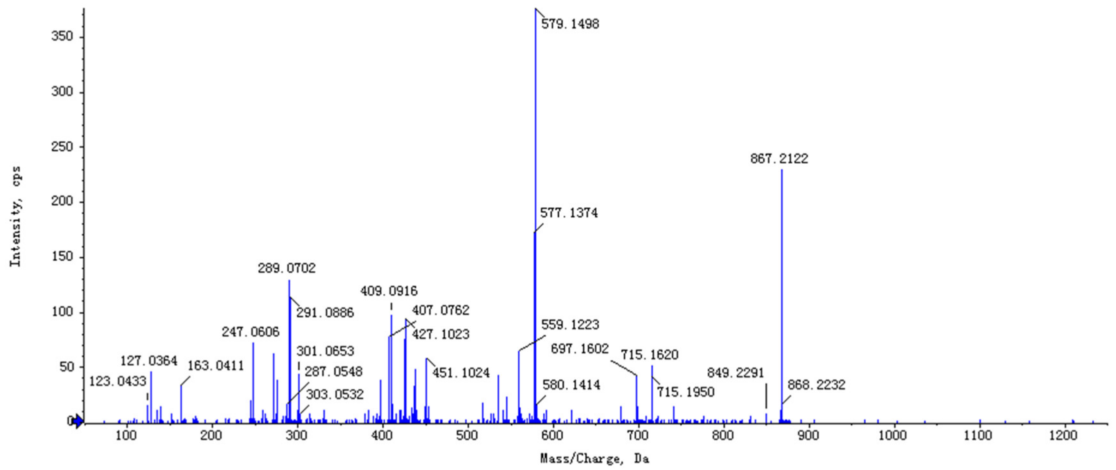

Figure S9 The MS/MS spectra of proanthocyanidin C1 obtained from the library construction
